# Technique and results of novel intracorporeal “overlap” colorectal anastomosis for laparoscopic and robotic surgery

**DOI:** 10.1007/s10151-026-03308-6

**Published:** 2026-04-30

**Authors:** A. V. Sazhin, I. V. Ermakov, G. B. Ivakhov, I. S. Lebedev, K. D. Dalgatov, M. V. Poltoratsky, I. S. Shikhin, N. A. Timoshenko, I. A. Morozov

**Affiliations:** 1 Pirogov Russian National Research Medical University (Pirogov University), Ostrovitianov St., 1, Moscow, 117513 Russia; 2 Moscow Multidisciplinary Clinical Center “Kommunarka” (MMCC “Kommunarka”), Sosensky Stan Str., 8, Moscow, 108814 Russia

**Keywords:** Colorectal neoplasms, Laparoscopy, Robotic surgical procedures, Intracorporeal anastomosis, “Overlap” anastomosis, Anterior resection

## Abstract

**Background:**

Laparoscopic colorectal procedures are widely adopted for colorectal cancer surgery. The double-stapling technique for circular colorectal anastomosis carries leakage rates of 11.2–13.4% and elevates stricture risk. In 2024, we introduced an innovative intracorporeal linear isoperistaltic “overlap” colorectal anastomosis; preliminary data from ten patients confirmed its safety and feasibility. This study aims to assess the feasibility and safety of the colorectal “overlap” anastomosis technique for laparoscopic and robotic surgery on a larger group of patients.

**Methods:**

An observational study was conducted from 2023 to 2025. A total of 100 patients with adenocarcinoma of the distal sigmoid colon, rectosigmoid junction, or upper rectum underwent laparoscopic or robotic colorectal surgery with intracorporeal “overlap” colorectal anastomosis. Demographic, intraoperative, and postoperative data, including complications, length of hospital stay, and 30-day readmission rates, were analyzed. Colonoscopy at 6 months assessed anastomosis configuration and patency.

**Results:**

The intracorporeal linear “overlap” colorectal anastomosis was performed in 100 patients (51 laparoscopic, 49 robotic—da Vinci Xi). Mean age was 67.0 ± 10.1 years and median body mass index (BMI) 26.9 kg/m^2^ (interquartile range [IQR]: 24.4–30.4); 76 patients were classified as American Society of Anesthesiologists (ASA) II and 24 were ASA III. Per pTNM staging, 33 patients had stage I, 27 stage II, 38 stage III, and 2 stage IV. Median blood loss was 30.0 mL (20–50), operative time 240.0 min (210.0–282.5), lymph nodes harvested 13 (11–18), and time to bowel function recovery 48 h (24–48). No intraoperative complications, conversions, or technical deviations, anastomotic leaks, major complications (Clavien–Dindo grades ≥ III), strictures, or 30-day readmissions occurred. Median postoperative stay was 5 days (4–6).

**Conclusions:**

The novel intracorporeal linear “overlap” colorectal anastomosis is safe and feasible and may be recommended as a reliable alternative to the conventional circular anastomosis in both laparoscopic and robotic colorectal surgery.

**Supplementary Information:**

The online version contains supplementary material available at 10.1007/s10151-026-03308-6.

## Introduction

According to the World Health Organization, the global incidence of colorectal cancer (CRC) continues to rise [1]. Surgical procedures remain the cornerstone of radical treatment for CRC [2–4]. The double-stapling technique (DST) using circular staplers, first described by Knight and Griffen, is the conventional approach [5, 6]. Despite advancements in stapling technology and the adoption of laparoscopic and robotic surgical techniques, these minimally invasive approaches typically require an extracorporeal phase [7, 8]. The reported incidence of anastomotic leakage and strictures following colorectal surgery remains considerable, reaching up to 11.2% and 13%, respectively [7, 9, 10]. In 2010, Inaba et al. introduced a new method of intracorporeal esophagojejunostomy using a linear stapling device, termed the “overlap” method [11]. In 2024, we published the first report describing a novel intracorporeal linear isoperistaltic “overlap” colorectal anastomosis [12]. Our work aims to analyze our clinical experience and outcomes with the implementation of intracorporeal “overlap” colorectal anastomosis in laparoscopic and robotic-assisted colorectal surgery.

## Materials and methods

### Methods

Prior to enrollment, the benefits and limitations of the intracorporeal “overlap” colorectal anastomosis procedure were explained to all patients, and preoperative informed consent was obtained. All surgeries were performed by four experienced colorectal surgeons as leaders, each with a minimum of 20 annual cases involving right and left colon resections with intracorporeal anastomosis, by either laparoscopic or robotic approaches. Patients underwent standardized preoperative assessments, including routine blood tests and contrast-enhanced computed tomography (CT) of the chest, abdomen, and pelvis to rule out contraindications to surgery or the presence of distant metastasis. The institutional ethics committee approved the study protocol.

The inclusion criteria were: patients aged 18–90 years who provided informed consent to participate; American Society of Anesthesiologists (ASA) status I–III; Eastern Cooperative Oncology Group (ECOG) performance status 0–1; histologically confirmed adenocarcinoma of the distal sigmoid colon, rectosigmoid junction, or upper rectum; clinical stage cT1–T4; and scheduled for intracorporeal linear isoperistaltic “overlap” colorectal anastomosis. Tumor staging was determined according to the 8th edition of the TNM classification (2017). The authors state that all participating patients signed voluntary informed consent.

The exclusion criteria were: refusal to participate, history of left colon or rectal surgery, cases requiring left hemicolectomy, evidence of acute colonic obstruction, ASA class IV or greater, or ECOG performance status ≥ 2. Intraoperative parameters included conversions to open surgery, modifications to the anastomosis technique, estimated blood loss, and operative duration. Postoperative outcomes assessed included complication rates, length of hospital stay, and 30-day readmissions. Complications were classified according to the Clavien–Dindo classification [13].

Anastomotic leakage was defined according to the International Study Group of Rectal Cancer (ISREC) criteria: clinical symptoms (abdominal pain or tenderness, peritonitis, fever, tachycardia, purulent or fecal discharge from an abdominal drain), blood test results (elevated white blood cell count and/or C-reactive protein), and radiological signs of an interruption of the anastomosis and/or a perianastomotic collection on CT [14].

Functional outcomes were evaluated by colonoscopy 6 months postoperatively.

### Patient cohort

From October 2023 to June 2025, a prospective observational study was conducted. A total of 100 patients with adenocarcinoma of the distal sigmoid colon, rectosigmoid junction, or upper rectum underwent laparoscopic or robotic anterior resection with intracorporeal linear “overlap” colorectal anastomosis. Laparoscopic procedures were performed in 51 cases, and robotic-assisted techniques in 49. All patients received preoperative mechanical bowel preparation and antibiotic prophylaxis in accordance with current guidelines [15]. Of the entire cohort, 41 patients underwent colonoscopy assessment at 6 months postoperatively to evaluate functional results.

### Surgical technique

Patients were placed in the Trendelenburg position with lower limbs abducted, and the table was tilted to the right.

**Fig. 1:**
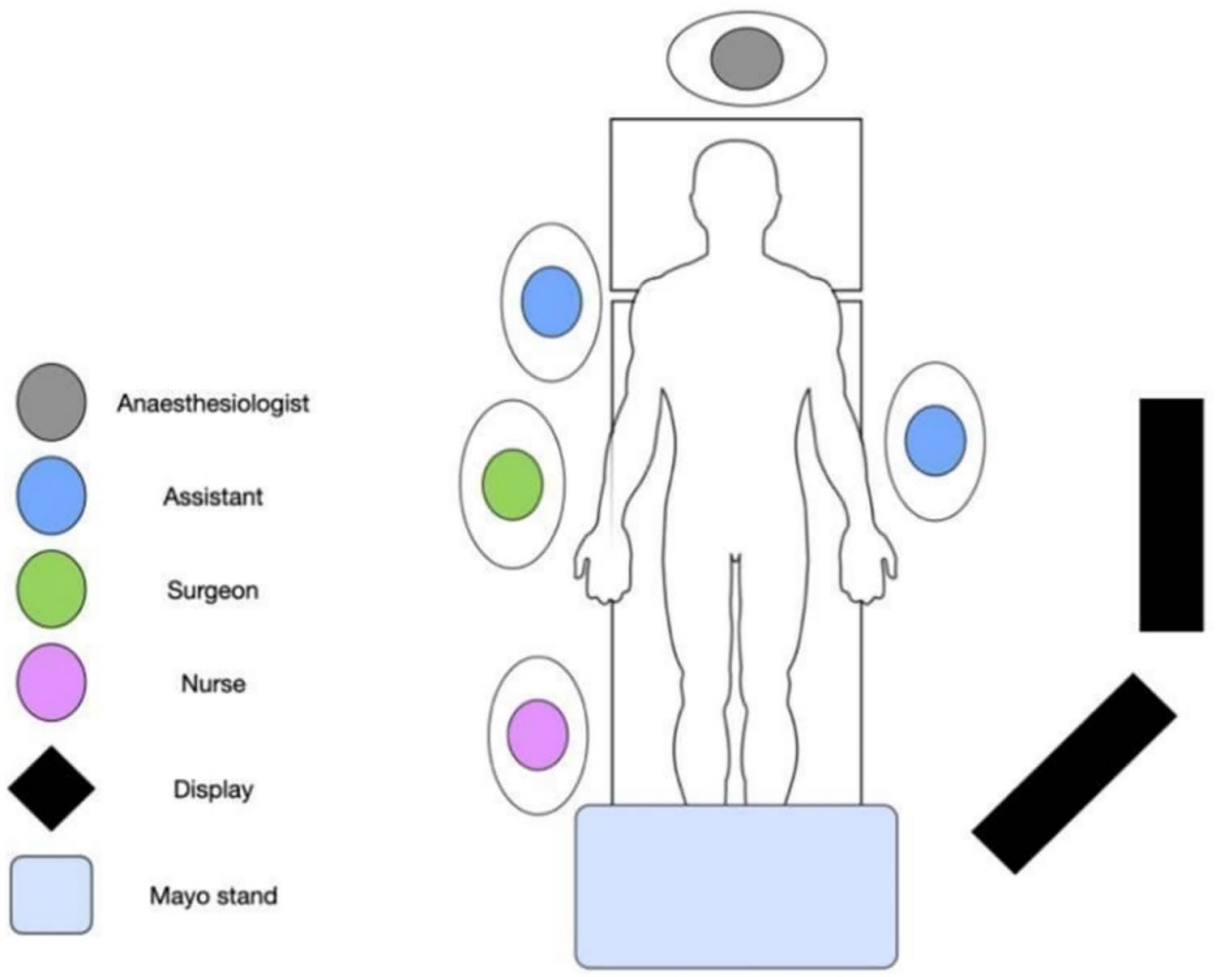
Position of surgical team during laparoscopic procedure

**Fig. 2:**
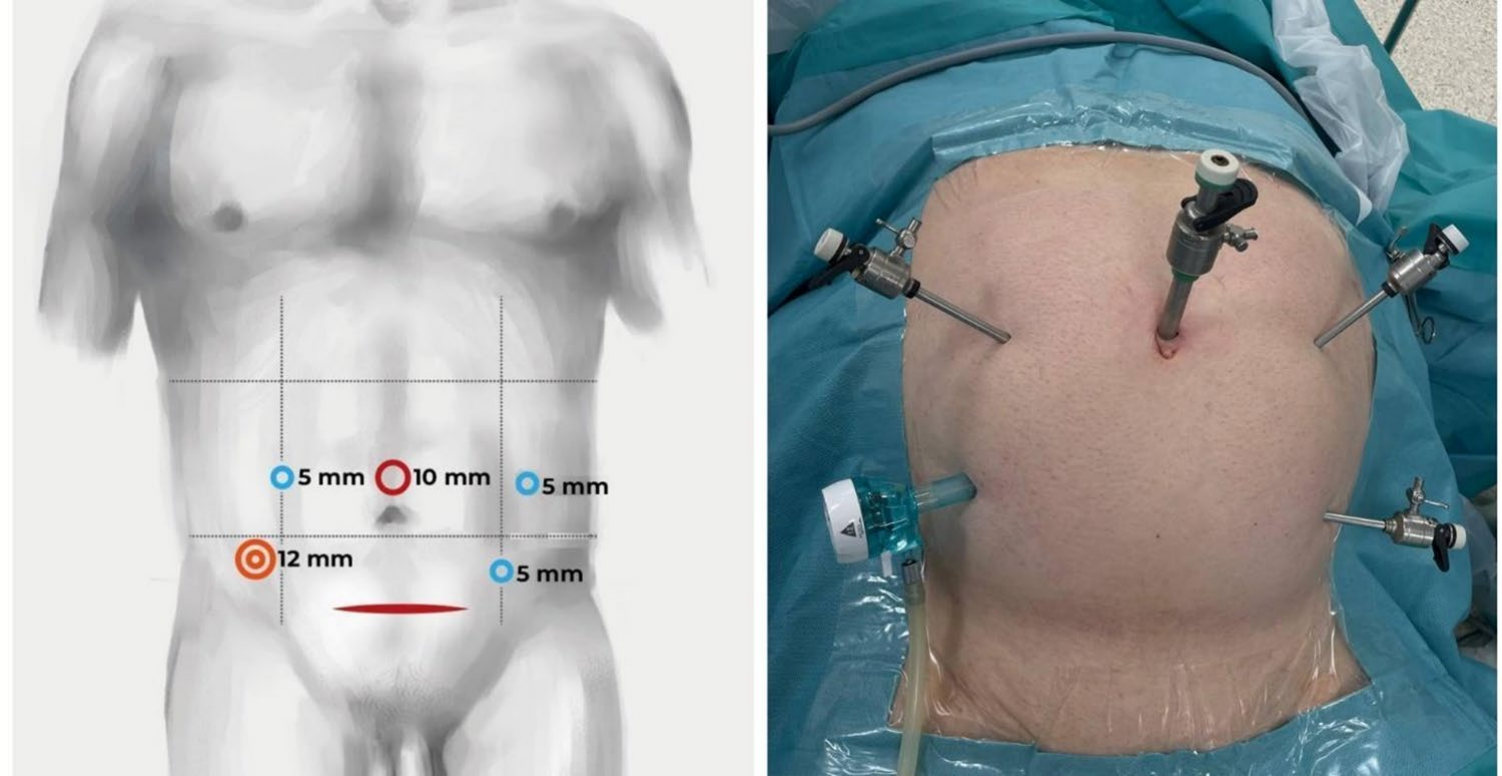
Trocar placement for laparoscopic intracorporeal “overlap” colorectal anastomosis

Laparoscopic colorectal resection with “overlap” anastomosis: The surgeon was stationed on the patient’s right, with the first assistant on the left and the camera assistant to the surgeon’s left (Fig. 1). Five trocars were typically used: a 10-mm supraumbilical port for the 30° laparoscope; a 12-mm port in the right lower quadrant for dissection, clipping, and stapling; and three 5-mm ports placed in the right upper and left lower abdominal quadrants (Fig. 2).

The procedure began with medial-to-lateral dissection and ligation of the inferior mesenteric artery (IMA) and inferior mesenteric vein (IMV). Surgeons performed either high or low ligation IMA on the basis of the surgeon’s preference. Complete mesocolic excision (CME) was performed, avoiding injury to Toldt’s fascia and the left ureter, and preserving the hypogastric nerves and pelvic parasympathetic plexus. The descending and sigmoid colon and upper rectum were mobilized, including partial mesorectal excision. Splenic flexure mobilization was selectively performed in cases of anticipated anastomosis tension.

**Fig. 3:**
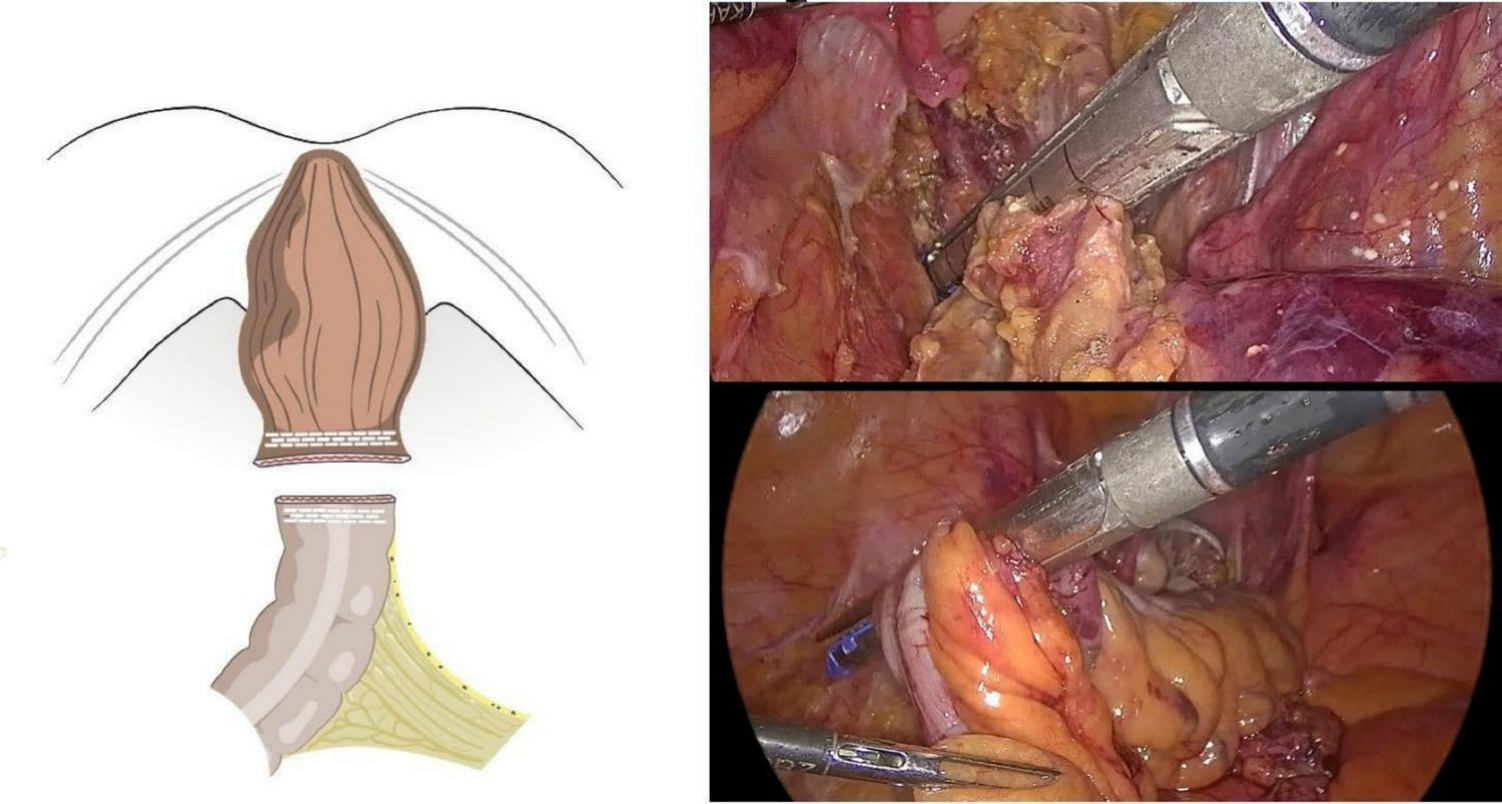
Bowel transection at the proximal and distal resection margins

Proximal and distal resection margins were defined intra-abdominally: For sigmoid colon cancer, the distal margin was ≥ 10 cm; for rectosigmoid or upper rectal cancers, it was ≥ 5 cm. The proximal margin was determined by the demarcation line following marginal artery clipping and extended ≥ 10 cm from the tumor. The mesocolon was dissected with ultrasonic shears, and lymphovascular pedicles managed accordingly; the intestine was cleared of adipose tissue and transected using a 60-mm linear stapler (blue cartridge) (Fig. 3). The resected specimen, including tumor, mesocolon, and lymphovascular pedicle, was immediately placed in an endobag.

**Fig. 4:**
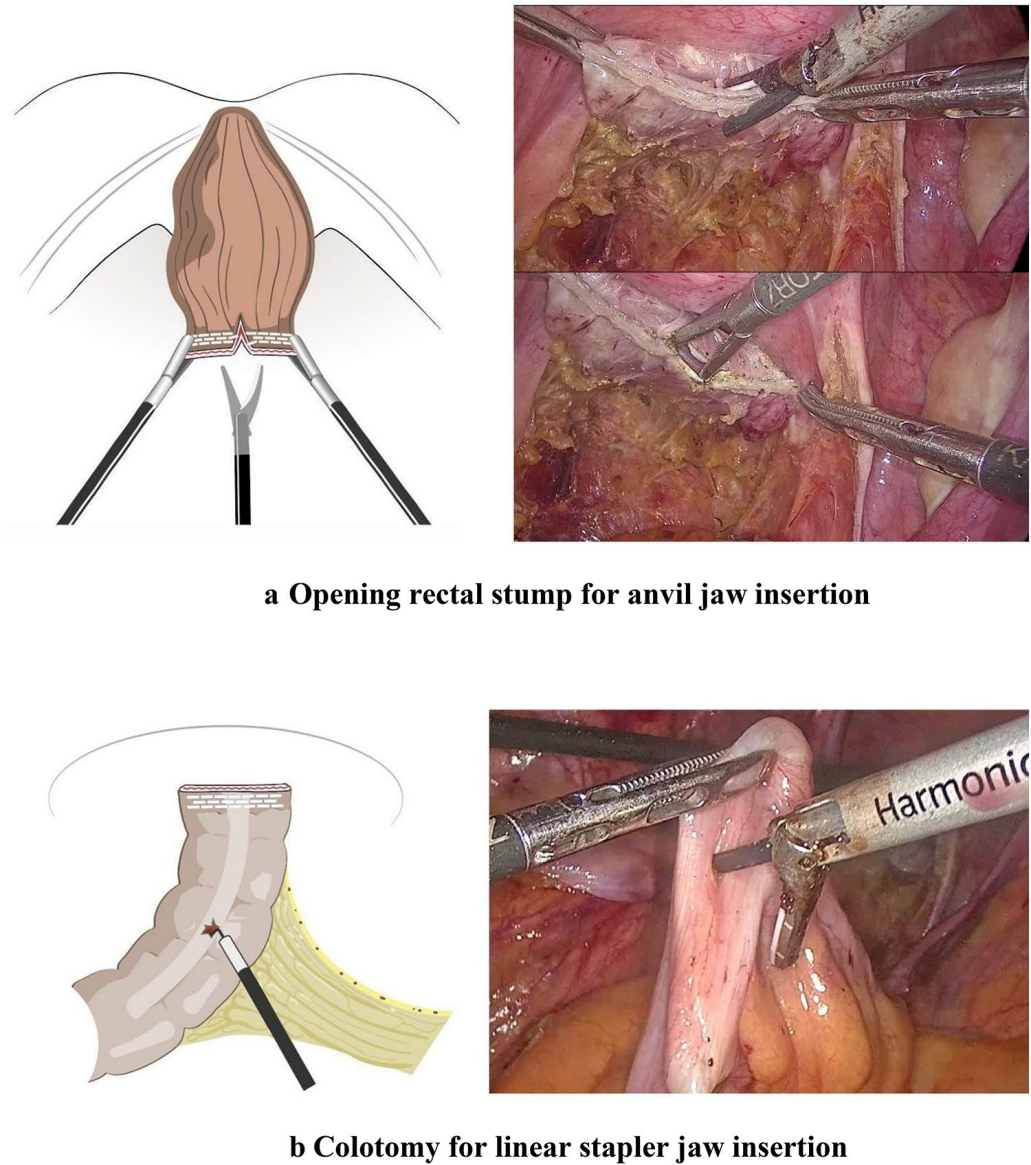
**a** Opening rectal stump for anvil jaw insertion. **b** Colotomy for linear stapler jaw insertion

**Fig. 5:**
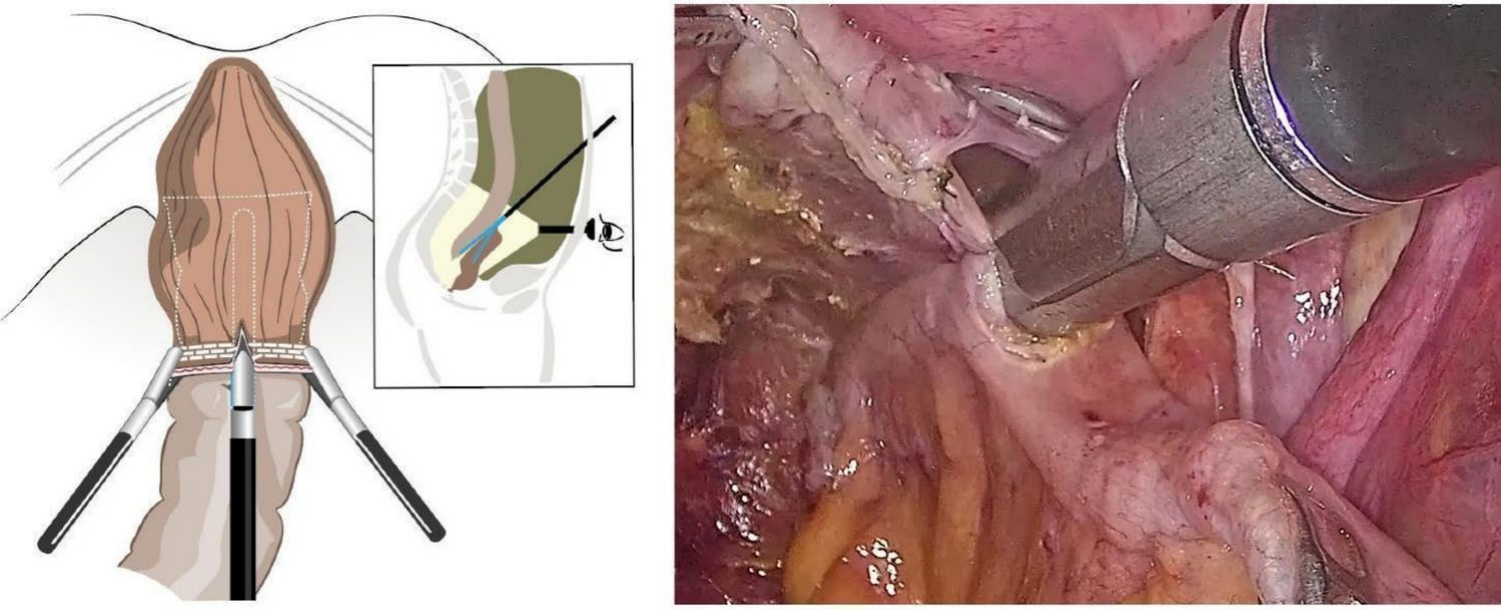
The formation of the intracorporeal linear “overlap” colorectal anastomosis

**Fig. 6:**
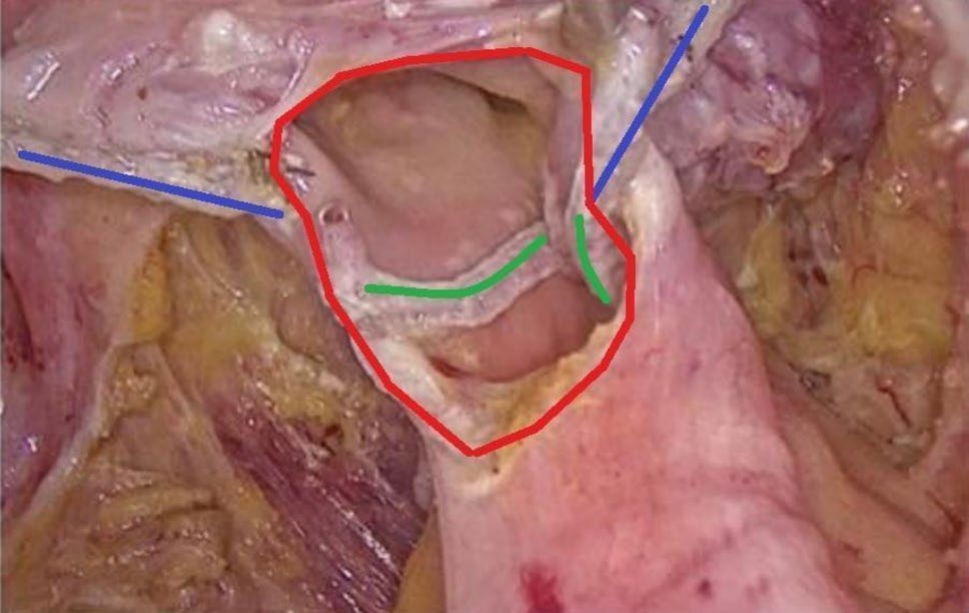
Interior view of the laparoscopic intracorporeal linear “overlap” colorectal anastomosis (blue lines—stapler suture line of the rectal stump; red line—entry hole; green lines—stapler line of the “overlap” anastomosis)

**Fig. 7:**
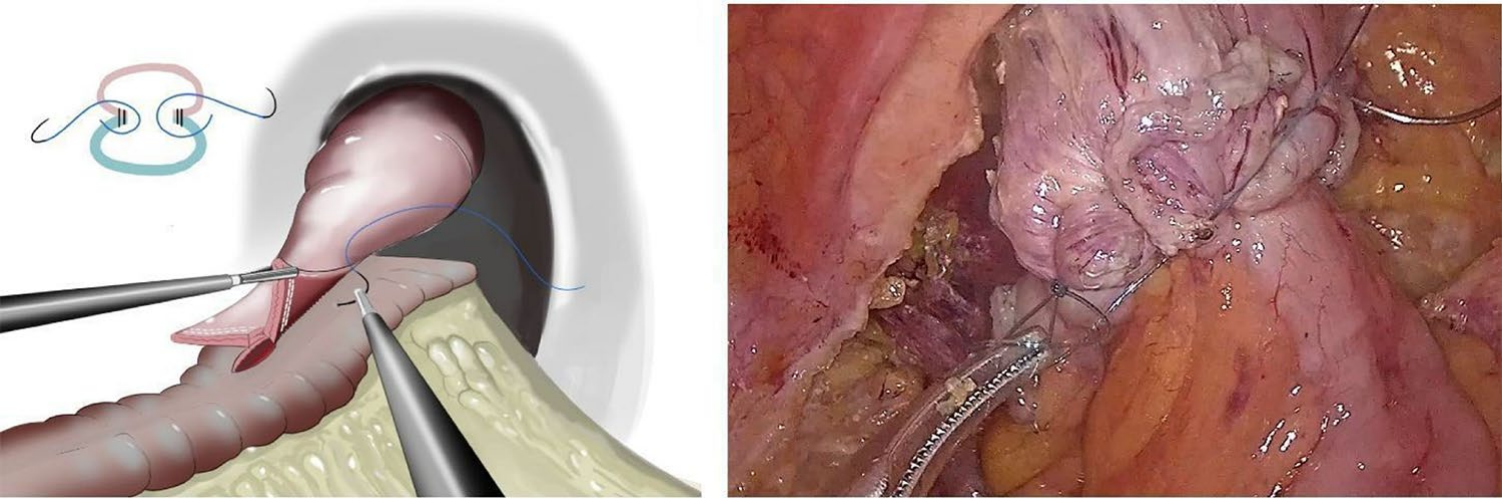
Entry hole closure

The rectal stump was retracted anteriorly and cranially. The posterior semicircle of the rectal stump was dissected with ultrasonic shears to facilitate stapler jaw placement for the “overlap” anastomosis. Prior to opening the rectal stump, the lumen was irrigated with 10% povidone-iodine via the anus. The stapler line of the rectal stump was divided in its mid portion using ultrasonic shears or laparoscopic scissors (Fig. 4a) under direct lumen and mucosal visualization to avoid submucosal tunneling. A colotomy was created on the antimesenteric border of the proximal stump, approximately 5 cm from its staple line, to facilitate insertion of the cartridge jaw (Fig. 4b). The jaws of a 45-mm linear stapler were inserted into the prepared colotomies, with the stapler’s suture line aligned along the posterior rectal wall. After closing and firing the stapler (Fig. 5), the common entry hole was closed transversely using a continuous single-layer 4–0 polydioxanone suture. Overlap between the hand-sewn suture and the stapler line was ensured at each end (Fig. 6–7). A bubble test was performed following the first suture row. Additional reinforcement with a second suture was at the discretion of the operating surgeon.

The specimen was retrieved via a transverse Pfannenstiel minilaparotomy using a wound protector and endobag. In female patients, vaginal specimen extraction via colpotomy was considered, with closure of the colpotomy performed using a continuous 2–0 Vicryl suture. An abdominal drain was placed, and all wounds were closed in layers.

**Fig. 8:**
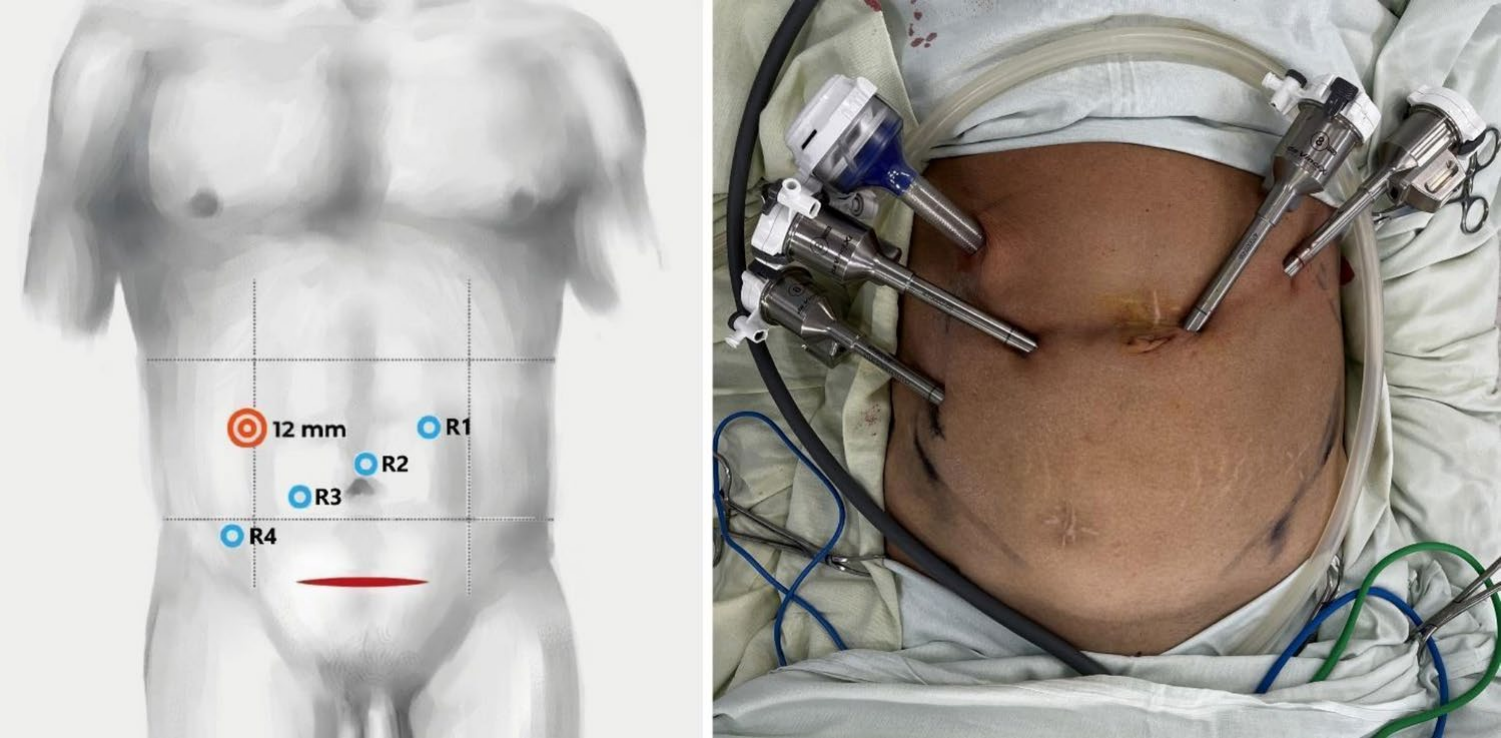
Trocar placement for a robot-assisted intracorporeal “overlap” colorectal anastomosis creation

For the robot-assisted “overlap” anastomosis technique, the da Vinci Xi system was used. The bedside assistant was stationed on the patient’s right side, and the robot was positioned on the left. Four robotic ports were placed diagonally to the right of the tumor, optimizing ergonomics for the da Vinci Xi system. A 12-mm assistant port was positioned in the right mesogastric region between arms R3 and R4 (Fig. 8).

All linear stapling was performed by the bedside assistant through the 12-mm port. The bowel was transected at proximal and distal resection margins with a 60-mm linear stapler. The resected specimen was placed in an endobag. Using robotic arms R1 and R4, the rectal stump was held cranially while R3 (equipped with scissors or the monopolar L-hook) dissected the posterior semicircle of the rectal stump free of mesorectal tissue, creating space for the stapler. Rectal irrigation and opening, as well as proximal colotomy, were performed as in the laparoscopic technique. Stapler insertion and firing were carried out by the bedside assistant, followed by closure of the common entry hole (as in the laparoscopic approach).

Laparoscopic and robotic-assisted “overlap” anastomosis techniques are demonstrated in short video files attached to this article.

### Statistics

Data were analyzed using Jamovi version 2.3.28 for MacOS (JMV 2.3.1 tool pack). Normality was assessed using the Shapiro–Wilk test. Quantitative variables with normal distribution (age) are presented as mean ± standard deviation (SD); non-normally distributed data (most of the continuous variables) are presented as median and interquartile range (IQR: Q1–Q3). Categorical variables were presented as frequencies (percentages).

## Results

A prospective analysis was performed on 100 patients. Patient demographics, preoperative characteristics, and pathological outcomes are presented in Tables 1, 2, 3, and 4. The laparoscopic group comprised 22 (43.1%) female and 29 (56.9%) male patients; and the robotic group 27 (55.1%) female and 22 (44.9%) male patients. The mean age of the cohort was 67.0 ± 10.1 years, with a median body mass index (BMI) of 26.9 kg/m^2^ (24.4–30.4). ASA classification was II in 76 and III in 24 patients. Per pTME staging, 33 (33%) patients were stage I, 27 (27%) stage II, 38 (38%) stage III, and 2 (2%) stage IV. Median intraoperative blood loss was 30 mL (20–50), and median operative duration was 240 min (210–282.5).

**Table 1:** Patient demographics

Parameter		All patients (*n* = 100)	Laparoscopic “overlap” (*n* = 51)	Robotic- assisted “overlap” (*n* = 49)
Sex	Male	51 (51%)	29 (56.9%)	22 (44.9%)
	Female	49 (49%)	22 (43.1%)	27 (55.1%)
ASA	II	76 (76%)	41 (80.4%)	35 (71.4%)
	III	24 (24%)	10 (19.6%)	14 (28.6%)
Age (years)^#^		67.0 ± 10.1	67.1 ± 10.6	66.8 ± 9.6
BMI, kg/m^2^^*^		26.9 (24.4–30.4)	26.5 (24.2–30.9)	27.1 (24.4–30.3)
Neoadjuvant chemotherapy		12 (12%)	7 (13.7%)	5 (10.2%)
Tumor site	Distal sigmoid	62 (62%)	31 (60.8%)	31 (63.3%)
	Rectosigmoid junction	25 (25%)	15 (29.4%)	10 (20.4%)
	Upper rectum	13 (13%)	5 (9.8%)	8 (16.3%)
Level of IMA ligation	High	50 (50%)	31 (60.8%)	19 (38.8%)
	Low	50 (50%)	20 (39.2%)	30 (61.2%)

^#^Data are presented as mean ± SD

^*^Data are presented as median [Q1; Q3]

**Table 2:** Perioperative data and outcomes

Parameter	All patients (*n* = 100)	Laparoscopic “overlap” (*n* = 51)	Robotic-assisted “overlap” (*n* = 49)
Postoperative complications	13 (13%)	9 (17.6%)	4 (8.2%)
Volume of blood loss, mL^*^	30 (20–50)	30 (20–50)	50 (30–50)
Duration of the operation, min^*^	240 (210–282.5)	215 (197.5–260)	255 (225–300)
First stool, h^*^	48 (24–48)	48 (24–48)	48 (24–48)
Length of stay (POD)^*^	5 (4–6)	5 (4–6)	5 (4–6)

^*^Data are presented as median [Q1; Q3]

**Table 3:** Postoperative complications (*n* = 13)

Clavien–Dindo grades	Types of complications	*n*	Total
I	Wound infection	3 (3%)	3 (3%)
II	Wound infection	4 (4%)	10 (10%)
	Deep-vein thrombosis	2 (2%)	
	Urinary retention or infection	2 (2%)	
	Pulmonary infection	1 (1%)	
	Arrhythmia and hypertension	1 (1%)	
III–V	No events	0	0

**Table 4:** Characteristics of examined specimens

Parameter			All patients (*n* = 100)	Laparoscopic “overlap” (*n* = 51)	Robotic-assisted “overlap” (*n* = 49)
Tumor size, cm^*^			3.5 (2.5–5.0)	3 (2.5–4.5)	3.5 (3.0–5.0)
Proximal resection margin, cm^*^		Colon	10.0 (10.0–10.0)	10.0 (10.0–10.0)	10.0 (10.0–11.0)
		Rectum	10.0 (10.0–11.5)	10.0 (10.0–15.0)	10.0 (10.0–10.0)
Distal resection margin, cm^*^		Colon	10.0 (10.0–10.0)	10.0 (10.0–10.0)	10.0 (10.0–10.0)
		Rectum	5.0 (5.0–9.3)	5.0 (5.0–10.0)	5.0 (5.0–6.8)
Lymph nodes harvested^*^			13 (11–18)	13 (11–18)	13 (11–18)
Stage (pTNM)	I		33 (33%)	17 (33.3%)	16 (32.7%)
	II		27 (27%)	16 (31.4%)	11 (22.4%)
	III		38 (38%)	16 (31.4%)	22 (44.9%)
	IV		2 (2%)	2 (3.9%)	0

^*^Data are presented as median [Q1; Q3]

**Fig. 9:**
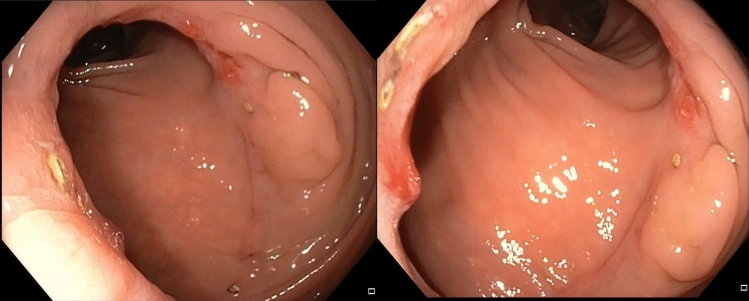
Endoscopy of the anastomosis 30 days after surgery

No intraoperative complications, conversions to open surgery, or changes in anastomotic technique occurred. The median number of lymph nodes retrieved was 13 (11–18), and bowel function returned at a median of 48 h (24–48) after first stool passage. Postoperatively, complications occurred in 13 patients (13%), 10 of whom developed fever, tachycardia, leukocytosis, and elevated C-reactive protein levels. To exclude anastomotic leakage per ISREC criteria, these patients underwent contrast-enhanced CT proctography, which ruled out intra-abdominal abscesses or anastomotic leaks. All complications were Clavien–Dindo grades I–II (13%), and no major complications (grades III–V) occurred. The median postoperative hospital stay was 5 days (4–6), with no 30-day readmissions. Of the 100 patients, 41 underwent 6-month follow-up colonoscopy, revealing no anastomotic strictures (Fig. 9).

## Discussion

Since its introduction in 1990, laparoscopic surgery has become widely adopted in colorectal procedures [16]. However, fully laparoscopic and robotic approaches for distal colon and rectal surgery remain limited [17]. The standard DST employs a circular stapler, including an extracorporeal phase, particularly to secure the anvil in the proximal bowel stump [5, 6]. Consequently, the main step of a minimally invasive procedure is interrupted, and in the case of robot-assisted surgery, redocking of the robotic platform is required. Extracorporeal anastomosis still requires a minilaparotomy, thereby discontinuing the main step of the procedure. An alternative may be the natural orifice specimen extraction surgery (NOSES) approach. According to current NOSES guidelines, there are some limitations related to the patient’s weight, sex, age, and tumor stage [18].

The DST carries notable drawbacks. Despite decades of experience, anastomotic leakage remains common (11.2–13.4%) [7, 19], primarily at staple intersections between circular and linear staplers [10]. Experimental studies have shown that DST anastomoses are inferior to linear staplers in terms of tensile strength [10, 20]. Smaller circular staplers (< 31 mm) are associated with higher stricture rates [21]. These issues have prompted investigation into alternative anastomotic techniques, although most studies emphasize leakage prevention or suture reinforcement rather than novel approaches [7, 22, 23].

The first reports of intracorporeal anastomosis using linear staplers appeared in 2002 [24]. The technique of intracorporeal anastomosis in laparoscopic right colectomy was standardized and further refined by Lechaux D. et al. in 2005, establishing its role in minimally invasive colorectal procedures [25]. In 2010, Inaba et al. presented a new method of the intracorporeal “end-to-side” esophagojejunostomy anastomosis using a linear stapling device, known as the “overlap” method [11]. There is ongoing controversy regarding the classification of “overlap” anastomosis as a “side-to-side” technique. Several studies describing intracorporeal anastomosis refer to the technique as the “overlap” method [26, 27]. However, on the basis of the detailed descriptions and schematic illustrations provided, these procedures align more closely with a true “side-to-side” anastomosis. We define true “overlap” anastomosis by direct incorporation of the staple line stump into the anastomosis.

In 2024, we described the intracorporeal linear isoperistaltic “overlap” colorectal anastomosis, initially reporting favorable safety and patient satisfaction in ten cases [12]. The use of intracorporeal linear “overlap” colorectal anastomosis, performed below the sacral promontory (S1), combines the advantages of intracorporeal anastomosis with the ability to achieve complete surgical resection through laparoscopic or robotic techniques. Our analysis of 100 cases shows that this technique is reproducible and safe. No intraoperative complications, conversions, or modifications of the anastomotic technique occurred, supporting its technical feasibility and safety. Intracorporeal techniques allow minilaparotomy at any site and offer specimen extraction via transvaginal route (NOSES), with wound size dictated by specimen size only.

Eliminating the extracorporeal stage reduces mesenteric tension and trauma, decreasing the risks of bleeding and intestinal ischemia — an important advantage, especially in patients with obesity [28]. The “overlap” technique prevents staple line intersection, uses a three-row stapler (potentially improving tensile strength and reducing leakage), and employs a “side-to-side” configuration, which further reduces complications, given that “end-to-end” anastomoses are linked to higher leakage [10, 29]. Owing to the complex shape of the entry hole, manual suturing closure is recommended. This technique is particularly well-suited for robotic surgery, enabling the entire anastomosis to be performed intracorporeally without undocking the robotic system.

## Conclusions

The novel intracorporeal linear “overlap” colorectal anastomosis is safe and feasible and may be recommended as a reliable alternative to the conventional circular anastomosis in both laparoscopic and robotic colorectal surgery.

## Supplementary Information

Below is the link to the electronic supplementary material.

Supplementary file1 (MP4 286471 KB)

Supplementary file2 (MP4 217214 KB)

## Data Availability

The data that support the findings of this study are not openly available owing to reasons of confidentiality and are available from the corresponding author upon reasonable request. Data are located in controlled access data storage at Pirogov Russian National Research Medical University.
